# Response of microbial diversity and function to the degradation of Barkol Saline Lake

**DOI:** 10.3389/fmicb.2024.1358222

**Published:** 2024-05-09

**Authors:** Yong-Hong Liu, Lei Gao, Hong-Chen Jiang, Bao-Zhu Fang, Yin Huang, Li Li, Shuai Li, Rashidin Abdugheni, Wen-Hui Lian, Jing-Yi Zhang, Zhen-Dong Yang, Osama Abdalla Abdelshafy Mohamad, Wen-Jun Li

**Affiliations:** ^1^State Key Laboratory of Desert and Oasis Ecology, Key Laboratory of Ecological Safety and Sustainable Development in Arid Lands, Xinjiang Institute of Ecology and Geography, Chinese Academy of Sciences, Urumqi, China; ^2^Xinjiang Key Laboratory of Biodiversity Conservation and Application in Arid Lands, Xinjiang Institute of Ecology and Geography, Chinese Academy of Sciences, Urumqi, China; ^3^State Key Laboratory of Biocontrol and Guangdong Provincial Key Laboratory of Plant Resources, School of Life Sciences, Sun Yat-Sen University, Guangzhou, China; ^4^School of Architecture and Civil Engineering, Chengdu University, Chengdu, China; ^5^Department of Biological, Marine Sciences and Environmental Agriculture, Institute for Post Graduate Environmental Studies, Arish University, Arish, Egypt

**Keywords:** saline lake, degradation, microbial community, diversity, ecological function

## Abstract

Barkol Lake, a shrinking hypersaline lake situated in the northeast of Xinjiang, China, has experienced the exposure of its riverbed and the gradual drying up of its original sediment due to climate change and human activities, resulting in the formation of alkaline soils. These changes have correspondingly altered the physicochemical characteristics of the surrounding environment. Microorganisms play a crucial role, with special functioning involved in various nutrient cycling and energy transfer in saline lake environments. However, little is known about how the microbial community dynamics and metabolic functions in this shrinking saline lake relate to the degradation process. To address this knowledge gap, a cultivation-independent method of amplicon sequencing was used to identify and analyze the microbial community and its potential ecological functions in the sediment and degraded area. The microbial community diversity was found to be significantly lower in the degraded areas than in the sediment samples. The *Pseudomonadota* was dominant in Barkol Saline Lake. The abundance of *Desulfobacterota* and *Bacillota* in the degraded areas was lower than in the lake sediment, while *Pseudomonadota*, *Acidobacteriota*, and *Actinobacteriota* showed an opposite trend. The βNTI showed that microbial community assembly was primarily associated with deterministic processes in Barkol Saline Lake ecosystems and stochastic processes at the boundary between sediment and degraded areas. Functional predictions showed that sulfur metabolism, particularly sulfate respiration, was much higher in sediment samples than in the degraded areas. Overall, these findings provided a possible perspective for us to understand how microorganisms adapt to extreme environments and their role in saline lakes under environmental change.

## Background

Lake degradation is a pressing environmental issue globally that leads to the deterioration of the ecological health and functioning of lakes, including biodiversity loss and water quality decline because of pollution, eutrophication, overexploitation of the water (Smith and Schindler, 2009; Schindler et al., 2016), biological invasion (Vanni et al., 2017), habitat destruction (Jeppesen et al., 1997), and climate change (Lu et al., 2013). Studies have shown that lake degradation often leads to the loss of native species and shifts in the community composition of freshwater organisms, aquatic plants, and invertebrates (Epners et al., 2010). Remote sensing analysis revealed that the major nine lakes in Central Asia declined in area dramatically from 9.14 × 10^4^ km^2^ to 4.60 × 10^4^ km^2^ from 1975 to 2007, and approximately half of the lakes were degraded to grassland or desert (Bai et al., 2011). Relevant research showed that lake degradation, when the lake dried up and the original sediment became alkaline soils, had an impact on soil physicochemical properties: soil water content decreased but pH increased, and total carbon and total nitrogen contents were significantly changed. The analysis of high-throughput sequences showed that the relative abundance of *Crenothrix* and *Methylocaldum* was decreased, while that of *Methylococcus* was increased due to lake degradation. The proportion of *Methylococcus* increased from 19.2 to 48.8 and 78.3%, respectively, while *Crenothrix* decreased from 54.7 to 32.1 and 13.9%, respectively, in the succession process of lake degradation to alkaline land and grassland (Mo et al., 2019).

Xinjiang is the largest province in China, located in the center of Eurasia, with a huge area of saline and alkaline soil, which is called the World Salt Soil Museum. However, most of the saline lakes are gradually drying or becoming salinized due to climate change and human activities. Barkol Lake is situated northeast of the Xinjiang Tianshan Mountains and is a shrinking sulfate-chloride-type hypersaline lake (Lu et al., 2012). As a typical continental saline lake, the basin area of Barkol Lake is approximately 4,514 km^2^, which is crucial to the stability of the surrounding ecosystem. Nevertheless, it has sharply shrunk during the last two decades, with a surface area of less than 90 km^2^ and an average depth of water of 0.6 m (Chen et al., 2019). Studies have shown that the microbial community in onshore soil responds differently to changes in geochemistry and mineralogy over time in the Aral Sea (Jiang et al., 2021). This indicates that the microbial community is highly sensitive to environmental changes and can provide valuable insights into the ecological health of the area. Furthermore, previous research conducted on different saline lakes in Xinjiang has revealed that the prokaryotic microbial community and potential biogeochemical cycles were influenced by the specific habitats of lakes (Liu et al., 2023). This highlights the importance of understanding the microbial community in saline lakes.

Therefore, the study of microbial communities in saline lakes is essential for assessing the impact of environmental changes and human activities on these ecosystems. This will provide valuable perspectives for the conservation and management of these unique and fragile environments, as well as help to understand the microbial community and potential ecological functions. However, knowledge of how microbial community composition and function respond to the degradation processes of Barkol Lake is still rare. We hypothesized that the degradation of the saline lake would affect the microbial communities and further impact the overall microecosystem on the ecological functions of carbon, nitrogen, sulfur, and other chemical elements. The main objective of this study is to: (1) investigate how the degradation of Barkol Saline Lake affects the microbial communities and their ecological functions and (2) provide an extensive understanding of the ecological roles of microorganisms from the degradation process of the saline lake.

## Materials and methods

### Sampling and physicochemical characterization

**Figure 1:**
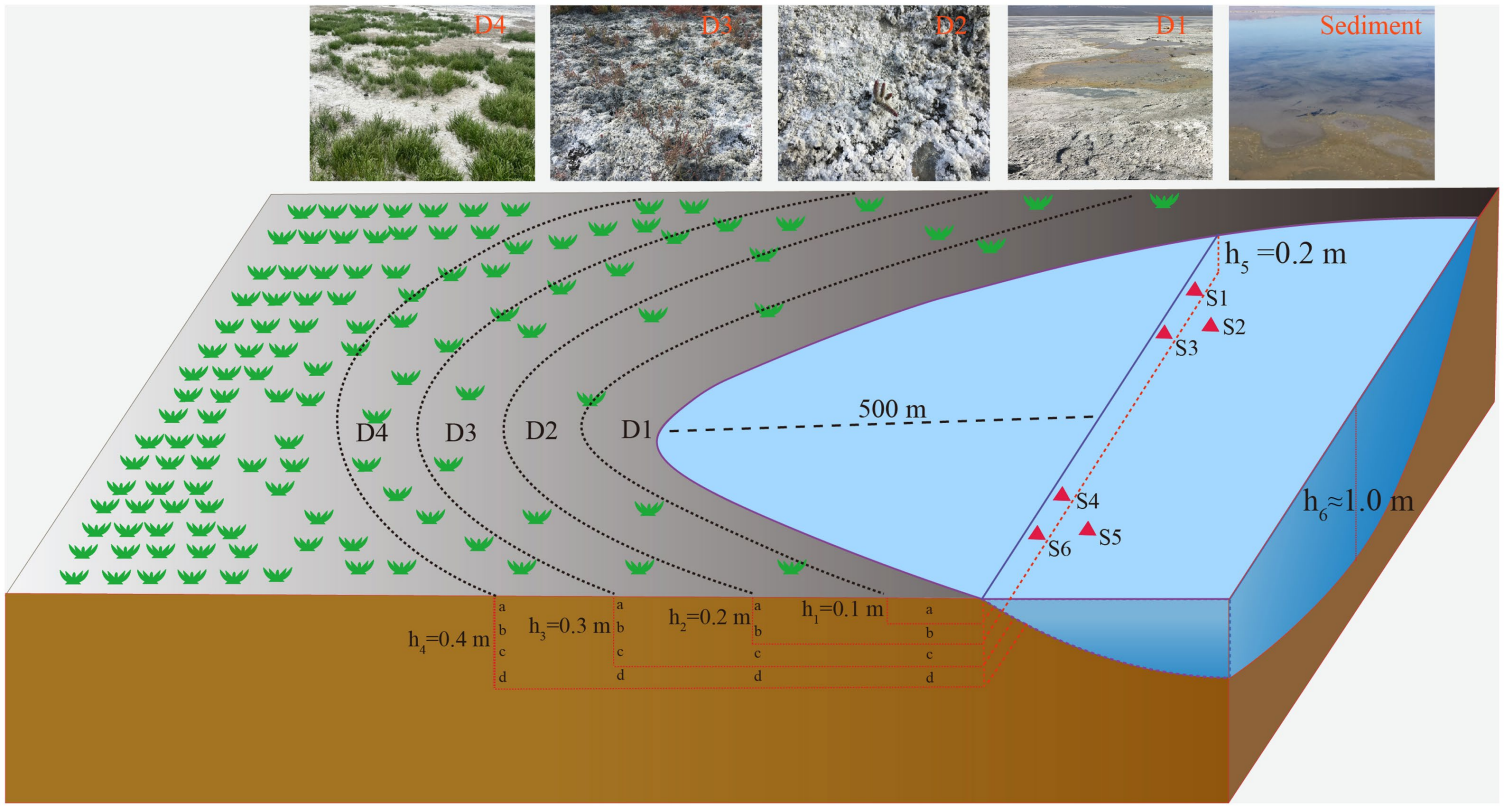
Schematic sampling sites show the location of sediments, and soil samples from the degraded area. S1, S2, S3, S4, S5, and S6 represent the samples from sediment, D1, D2, D3, and D4 represent the soil samples from degraded area.

A total of 34 samples, including18 samples of saline lake sediment and 16 samples of soil from the degradation area were collected from Barkol Saline Lake (Xinjiang, China, 92.8E; 43.6 N) by a geotome (The length is approximately 100 cm and the diameter is approximately 3.8 cm) on 12 July 2022. The sediment samples were obtained from the top 20 cm (at 70 cm depth below the water surface) at six different sites labeled S1 to S6 in triplicate at each site. The soil samples from the degraded area, were collected from four sites representing different stages of degradation defined by different degrees of salinization and plant coverage (primary degradation stage: D1; intermediate degradation stage: D2; severe degradation stage: D3; extreme degradation stage: D4) (Figure 1). These samples were taken at depths of 0–40 cm with intervals of 10 cm at each site. For example, D1a represents the sample from site 1 at a depth of 10 cm, and D3d represents the sample from site 3 at a depth of 40 cm (Figure 1). The samples are separated into two parts, one for molecular samples and another for physicochemical samples. The samples for DNA extraction were stored in liquid nitrogen during transportation and stored at −80°C upon arrival in the laboratory until further analysis. In addition, the samples for physicochemical analysis of environmental parameters such as K^+^, Na^+^, Ca^2+^, Mg^2+^, SO_4_^2−^, Cl^−^, CO_3_^2−^, and HCO_3_^−^ were air dried at room temperature. They were then ground into a homogeneous powder and sieved through a 2 mm sieve (Liu et al., 2019).

### DNA extraction and illumina sequencing

Community DNA was extracted from a 0.5 g sample using the PowerSoil Kit (MP Biomedical, OH, USA) following the manufacturer’s direction. The extracted DNA was quantified using a NanoDrop™ 2000 (Thermo Fisher Scientific, Wilmington, DE, USA) and amplified the V3–V4 regions of bacteria and archaea by using 16S rRNA genes with a set of bar-coded primers (338F and 806R) (Caporaso et al., 2012). PCR amplification was performed in a 25 μL volume reaction mixture containing 25 ng of template DNA, 12.5 μL of PCR Premix, 2.5 μL of each primer, and PCR-grade water to adjust the volume (Li et al., 2021). The PCR products from each sample were pooled with equimolar concentrations and then sequenced on a NovaSeq 6,000 platform (Illumina Inc., USA) (250 bp paired-end reads). Paired-end reads were assigned to samples based on their unique barcode and truncated by cutting off the barcode and primer sequence, then merged using FLASH (version 1.2.11). For obtaining high-quality tags, the sequence data were filtered by using Trimmomatic (version 0.39), and denoising and chimera checking were accomplished by using the DADA2 (Callahan et al., 2016) tool in QIIME2 software (Bolyen et al., 2019) (version 2020.6).

### Statistical analysis

By default, a threshold of 0.005% was utilized to filter ASVs, resulting in the removal of a small fraction of all sequences. The ASV-based analysis was conducted to assess the alpha diversity of the samples. The Pielou, Richness, Shannon, and Simpson indexes were calculated using QIIME2,^1^ a bioinformatics software package for microbiome analysis. The results were then visualized using R software (version. 4.2.2). To evaluate the richness of the microbial communities in the samples, rarefaction curves were generated. These curves depict the relationship between the number of sequenced samples and the observed diversity. By plotting the rarefaction curves, we can determine if the sequencing depth is sufficient to capture the full diversity of the microbial community or if additional sequencing is required. The beta diversity of the samples was evaluated using principal coordinate analysis (PCoA) based on UniFrac metrics. Additionally, the significance of compositional differences between microbiomes from soil samples D1, D2, D3, and D4 was assessed using the PERMANOVA test, with a significance threshold set at a *p*-value of <0.05. The unweighted pair group method with arithmetic mean (UPGMA) cluster analysis. These analyses were performed using the amplicon sequence variant (ASV) table obtained from each sample.

### Microbial community analysis

The taxonomic identity was determined using the SILVA 138 database (Quast et al., 2013). The annotations of each ASV were assigned to different taxonomic levels, including phylum, class, order, family, and genus. This comprehensive annotation allowed for further analysis and interpretation of the taxonomic composition of the samples. The community assembly mechanism of stochastic and deterministic processes was disentangled using a statistical framework based on null models (Zhou and Ning, 2017). The β nearest-taxon index (βNTI) was calculated using the R packages “picante” (version 1.7) and “vegan” (version 2.5-2). This calculation involved performing a null model test on the βMNTD and RC Bray metrics. Based on the abundance and variation of each species in each sample, a Spearman rank correlation analysis was conducted. The default method was used for this analysis. The data were then screened to identify correlations that had a value greater than 0.1 and a *p*-value less than 0.05. These correlations were used to construct a correlation network.

### Function prediction

Functional annotations were performed using the FAPROTAX database (Louca et al., 2016), a comprehensive resource that provides functional annotations for prokaryotic taxa based on their known metabolic capabilities, to predict the main processes of carbon, nitrogen, sulfur biogeochemical cycling, and nutrition type of community. This information can contribute to a better understanding of the overall functioning of prokaryotic communities in Barkol Saline Lake ecosystems. The ecological functions were also predicted by BugBase (Ward et al., 2017), a database that can make accurate predictions about the functional traits of a particular species based on its taxonomic classification and other available information.

## Results

### 16S rRNA gene sequencing data and microbial diversity

**Figure 2:**
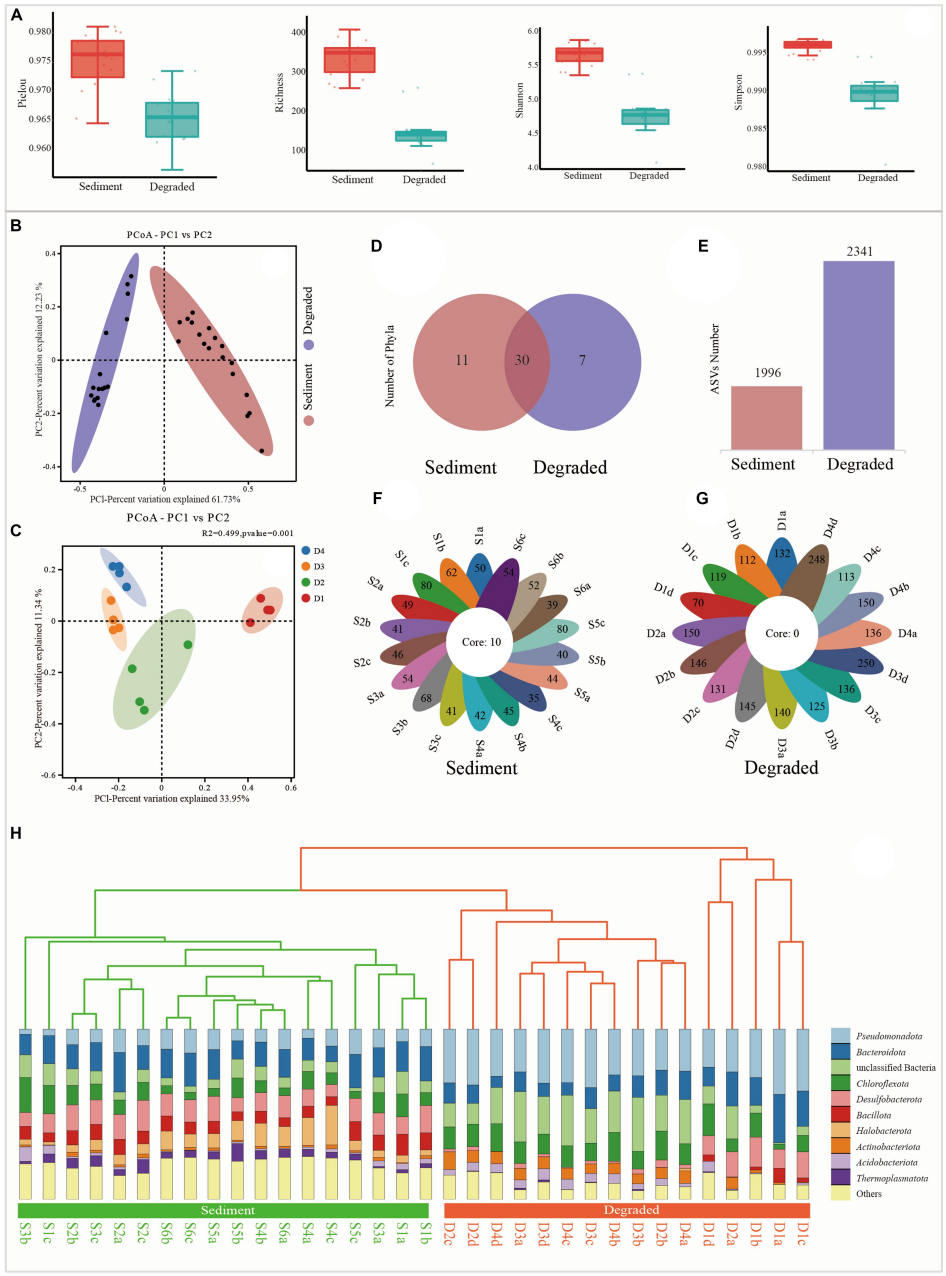
Alpha diversity and beta diversity indexes from sediment samples and degraded area samples. **(A)** Alpha diversity of Pielou, Richness, Shannon, and Simpson, respectively. **(B)** Principal coordinate analysis (PcoA) between sediment and degraded area soil, **(C)** principal coordinate analysis (PcoA) between soils from the different degraded processes of D1, D2, D3, and D4, **(D)** the distribution of Venn diagram at the phylum level, **(E)** ASVs number from the sediment and degraded area, **(F)** flower plot of all samples in the sediment, and the number on the flower plot lobes represents the unique ASVs from each sample, **(G)** flower plot of all samples in the degraded area, the number on the flower plot lobes represent the unique ASVs from each sample, and **(H)** unweighted pair-group method with arithmetic means (UPGMA).

A total of 2,134,301 clean reads were obtained from the 34 samples; each sample produced at least 36,283 clean reads, with an average of 62,774 clean reads per sample after removing low-quality sequences. The sequence length in this study was mostly distributed in the range of 350 bp. The alpha diversity indexes showed that the diversity of the microbial community was notably reduced from sediments to degraded areas of saline lake. Specifically, Pielou, Richness, Shannon, and Simpson indexes ranged from 0.965 to 0.981, 262 to 411, 5.4 to 5.9, and 0.994 to 0.997 in the sediment samples, but sharply dropped to 0.957 to 0.973, 70 to 263, 4.1 to 5.4, and 0.980 to 0.995 in the degraded area, respectively (Figure 2A; Supplementary Table S1). The sequence number, relative abundance, and read abundance are much more abundant than those in degraded areas (Supplementary Figure S1).

The UPGMA tree and PCoA analysis clearly showed that the 34 samples could be divided into two distinct groups, each representing a different prokaryote community with unique features. This indicated that there were significant differences between the microbial communities in the sediment samples and those in the degraded areas (Figures 2B,C,H). As shown from the Venn diagram and histogram (Figures 2D,E), the microbial groups in the sediment samples had a total of 1996 ASVs belonging to 41 phyla and 10 core groups (Figure 2F), while the degraded area samples had a total of 2,341 ASVs belonging to 37 phyla but no core group (Figures 2D,E,G).

### Microbial community composition and structure

**Figure 3:**
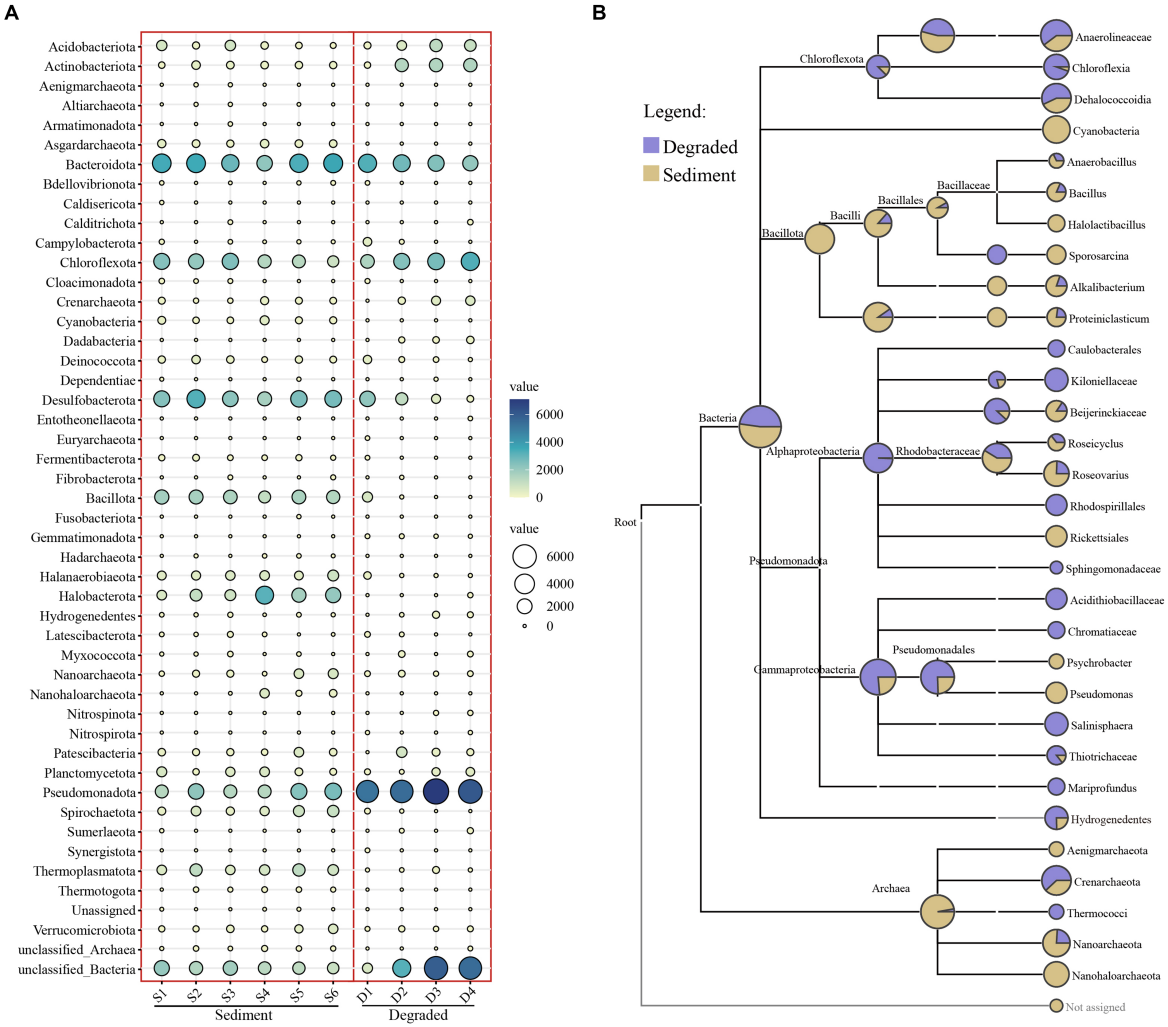
Bubble chart **(A)** at the phylum level and taxa tree **(B)** from the phylum to genus level of prokaryotic microbe from sediment and degraded area samples. The abundance of microbes was indicated by the size of the bubbles; in addition, the abundance of microbes was also indicated by colors: blue indicating high abundance and white indicating low abundance.

The taxonomic assignment of ASVs in the sediment of the saline lakes and soil from degraded areas revealed 47 different phyla, 90 classes, 197 orders, 321 families, and 445 genera (Supplementary Table S2). Among all phyla, *Pseudomonadota* were widely distributed in all kinds of samples with an average abundance of 18.87%, followed by *Bacteroidota* (14.91%), unclassified Bacteria (12.69%), *Chloroflexota* (10.85%), and *Desulfobacterota* (9.27%) (Supplementary Table S3). However, the community composition of prokaryotes varied between the sediment of the saline lake and the degraded area. The abundance of *Desulfobacterota*, *Bacillota*, *Halanaerobiaeota*, *Halobacterota*, *Spirochaetota*, *Thermoplasmatota*, and *Asgardarchaeota* decreased as the lake sediment degraded into soil; on the contrary, the abundance of *Pseudomonadota*, *Acidobacteriota*, and *Actinobacteriota* increased with the degradation process; meanwhile, the degradation process of the saline lake had not much effect on the abundance of *Bacteroidota*, *Chloroflexota*, *Patescibacteria*, and *Planctomycetota* in this study (Figure 3A). Furthermore, *Cyanobacteria*, *Nanohaloarchaeota*, and *Aenigmarchaeota* were unique or apparently high in the sediment samples of saline lake at the phylum level, whereas, *Mariprofundus*, *Caulobacterales*, and *Rhodospirillales*, were representative groups in the degraded area of saline lake at class and order level, respectively. As for the degraded area of the saline lake, the *Kiloniellaceae*, *Sphingomonadaceae*, *Acidithiobacillaceae*, and *Chromatiaceae* were unique groups at family level. However, *Halolactibacillus*, *Sporosarcina*, and *Psychrobacter* were only found in the sediment samples of the saline lake at the genus level (Figure 3B).

The relative abundance was much higher in sediment samples compared to soil samples from degraded areas, except Site 3d and Site 4d (Supplementary Figure S1), which indicated that the samples from degraded areas had far fewer microbial groups than those from sediment areas. In terms of specific microbial groups, the *Asgardarchaeota*, *Bacillota*, *Halanaerobiaeota*, *Halobacterota*, *Spirochaetota*, and *Thermoplasmatota* were representative groups mainly distributed in sediment environments. Additionally, there were many unclassified bacterial and archaeal groups in our samples, with a large proportion of ASVs not annotated at the phylum level. This may be that some species were not annotated due to the small size of the amplified fragments. Another possibility was that Barkol Lake habitat may harbor a significant number of potential novel microbial resources that require further exploration (Supplementary Figure S2).

### The co-occurrence network analysis

**Figure 4:**
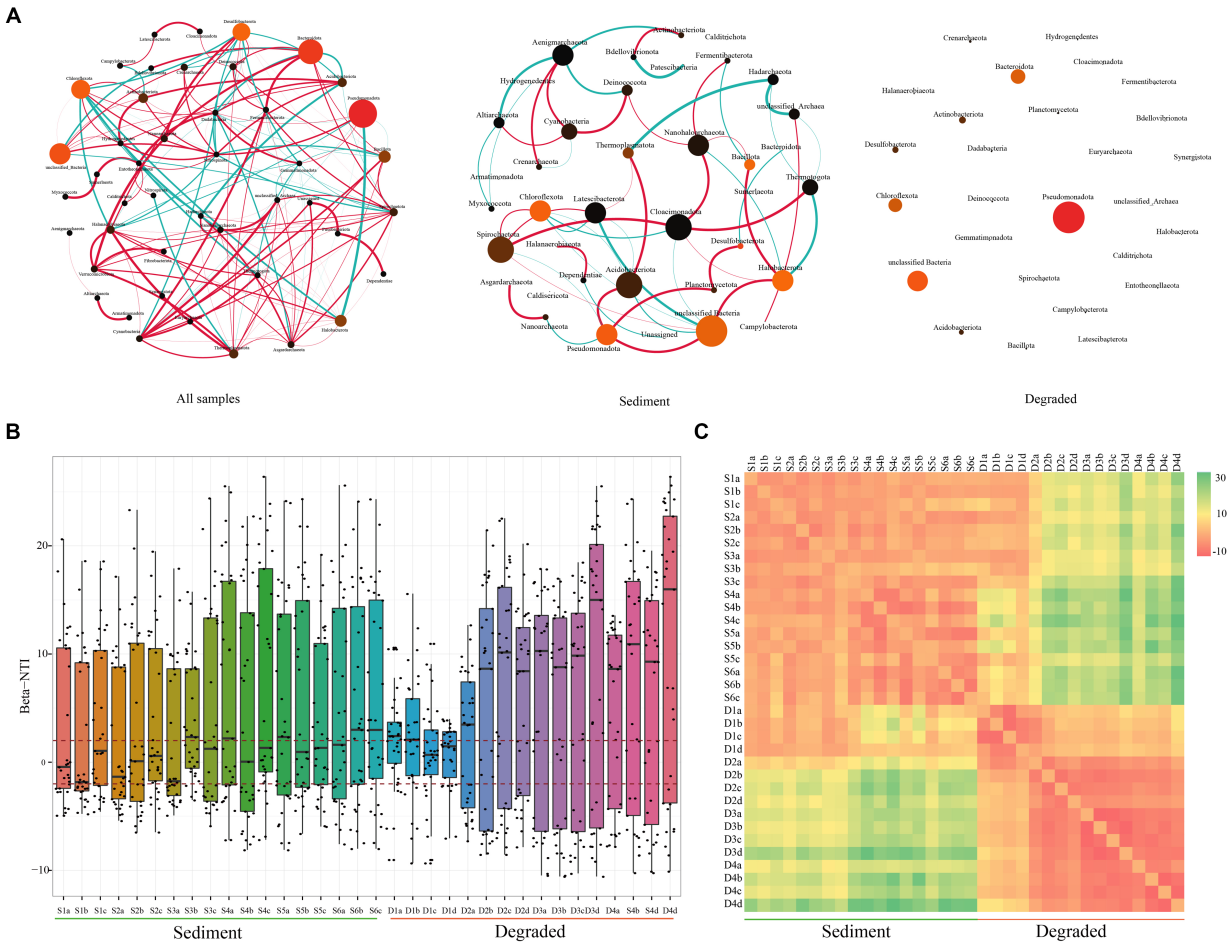
Overview of the community co-occurrence network analysis and βNTI analysis. **(A)** Network analysis of all samples, sediment samples, and soil samples from the degraded area. The correlation was distinguished by red (positive correlation) and green (negative correlation) of edges, and the degree of correlation was proportional to the thickness of the edges, the node size was proportional to the abundant of microbial taxa; **(B)** the beta nearest-taxon index (βNTI), and **(C)** the RC-bray to explore the relative roles of stochastic and deterministic processes in shaping microbial community assembly.

Based on the above findings, we speculated that the microbial community structure in sediments was stable, while that in degraded area samples was variable. To verify the conclusions, we constructed the co-occurrence network patterns of microbial from Barkol Saline Lake habitat using the ASVs from all samples, sediment samples, and also the samples from degraded area at the phylum level, respectively (Figure 4A). It revealed that the microbial community in the habitat of Barkol Saline Lake showed an intricate network relationship, in which different groups showed positive or negative correlations. For example, *Desulfobacterota* showed a strong positive correlation with *Nanoarchaeota* but a negative correlation with *Chloroflexota*, *Acidobacteriota,* and *Nitrospinota*. As we suspected, the unclassified Bacteria group appeared to have an important position in the sediment network, with connections to seven other groups. The relationship between *Spirochaetota* and other groups was predominantly positive. Nevertheless, with regard to degraded areas, the community showed no relationships with each other in the network from this study.

### The community assembly mechanism and driving factors in Barkol ecosystems

We used the βNTI to explore the relative roles of stochastic and deterministic processes in shaping microbial community assembly. The majority of the βNTI values among all the samples were higher than 2, suggesting that the microbial community assembly in Barkol Saline Lake ecosystems was primarily driven by deterministic processes, specifically variable selection (Figure 4B). This means that certain environmental factors or conditions are selecting for specific microbial taxa, leading to the observed community composition. However, there was an interesting observation at the boundary between sediment and degraded area. The βNTI value at the boundary between sediment and degraded area was less than 2, suggesting that the microbial community composition in this area was undergoing a stochastic process, which means that the bacterial community composition in this specific area was randomly formed and not significantly influenced by the external environment (Figures 4B,C). Overall, these findings highlight the importance of both deterministic and stochastic processes in shaping microbial community assembly in Barkol Saline Lake ecosystems.

### The physicochemical characteristics of the samples

**Figure 5:**
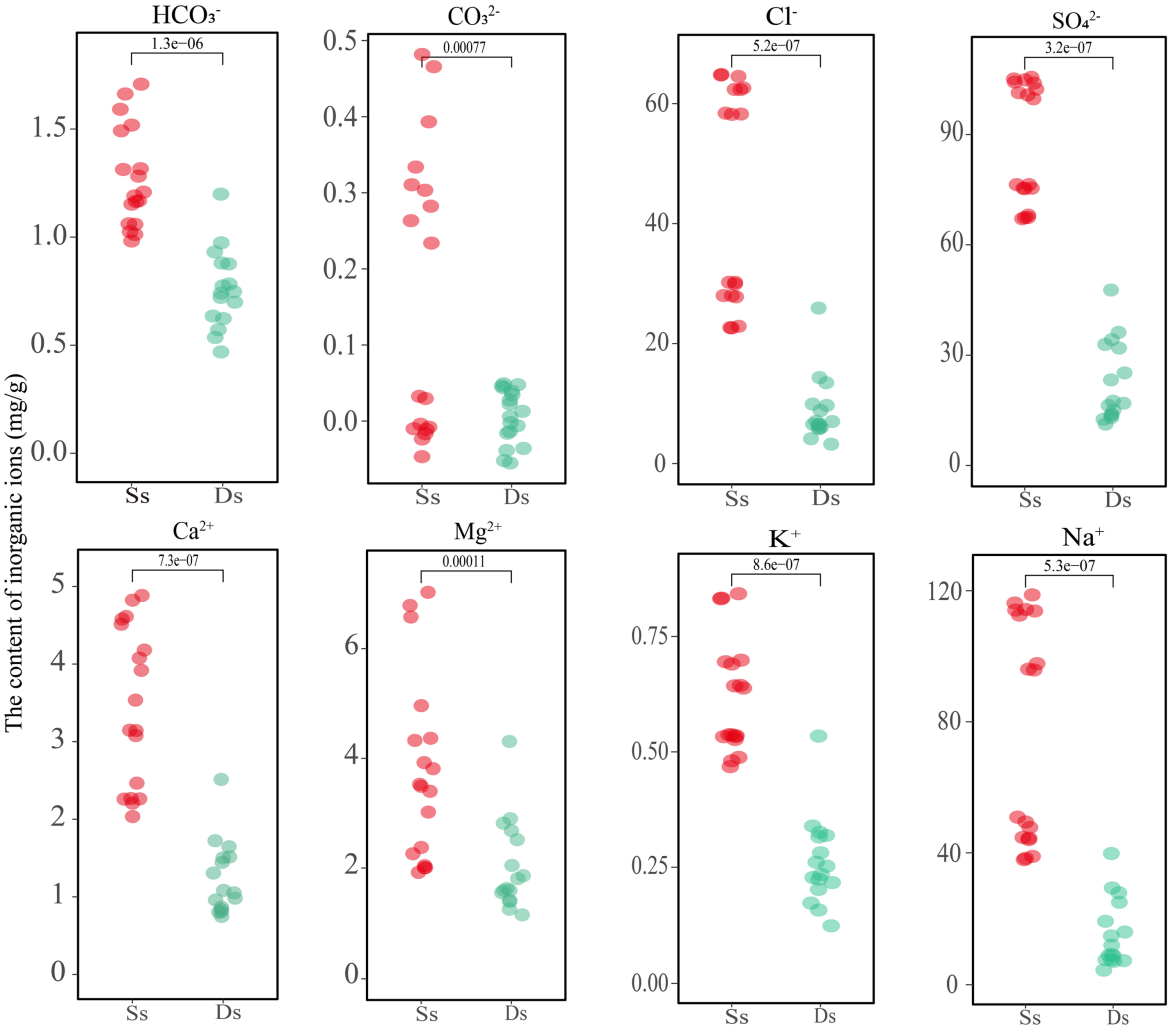
Analysis of inorganic ion content in sediment and soil of Barkol Lake (mg/g). Ss, sediment; Ds, degraded.

As shown above, deterministic processes play a dominant role in most areas, and further research was needed to understand the specific factors driving these processes and their implications for ecosystem functioning in Barkol Saline Lake. The physicochemical properties of the samples, including the inorganic ion components, are shown in Supplementary Table S4 and Figure 5. The samples denoted with an Ss are sediment and Ds are soils from degraded areas. The analysis revealed that the high concentration of Na^+^ and SO_4_^2−^ ions in the sediment of the sulfate-type salty lake indicated its strong salinity. Comparatively, the concentration of the four major anions CO_3_^2−^, HCO_3_^−^, Cl^−^, and SO_4_^2−^, as well as the four cations Ca^2+^, Mg^2+^, K^+^, and Na^+^ showed a decreasing trend after lake degradation (Figure 5). This indicates that the overall concentration of salt ions decreased as the lake degraded, which will eventually lead to a decrease of total salinity and inevitably change the corresponding microbial groups in this habitat. In addition, the content of inorganic ions carbonate ions (CO_3_^2−^) and bicarbonate ions (HCO_3_^−^) was relatively low in this habitat. A small amount of CO_3_^2−^ was detected in the sediment samples, but it was not detected in the soil samples from the degraded area, or its content was lower than the detected value. Further investigation would be needed to determine the exact source and implications of the CO_3_^2−^ in the sediment samples.

### The distribution of the representative phylum *Desulfobacterota*

**Figure 6:**
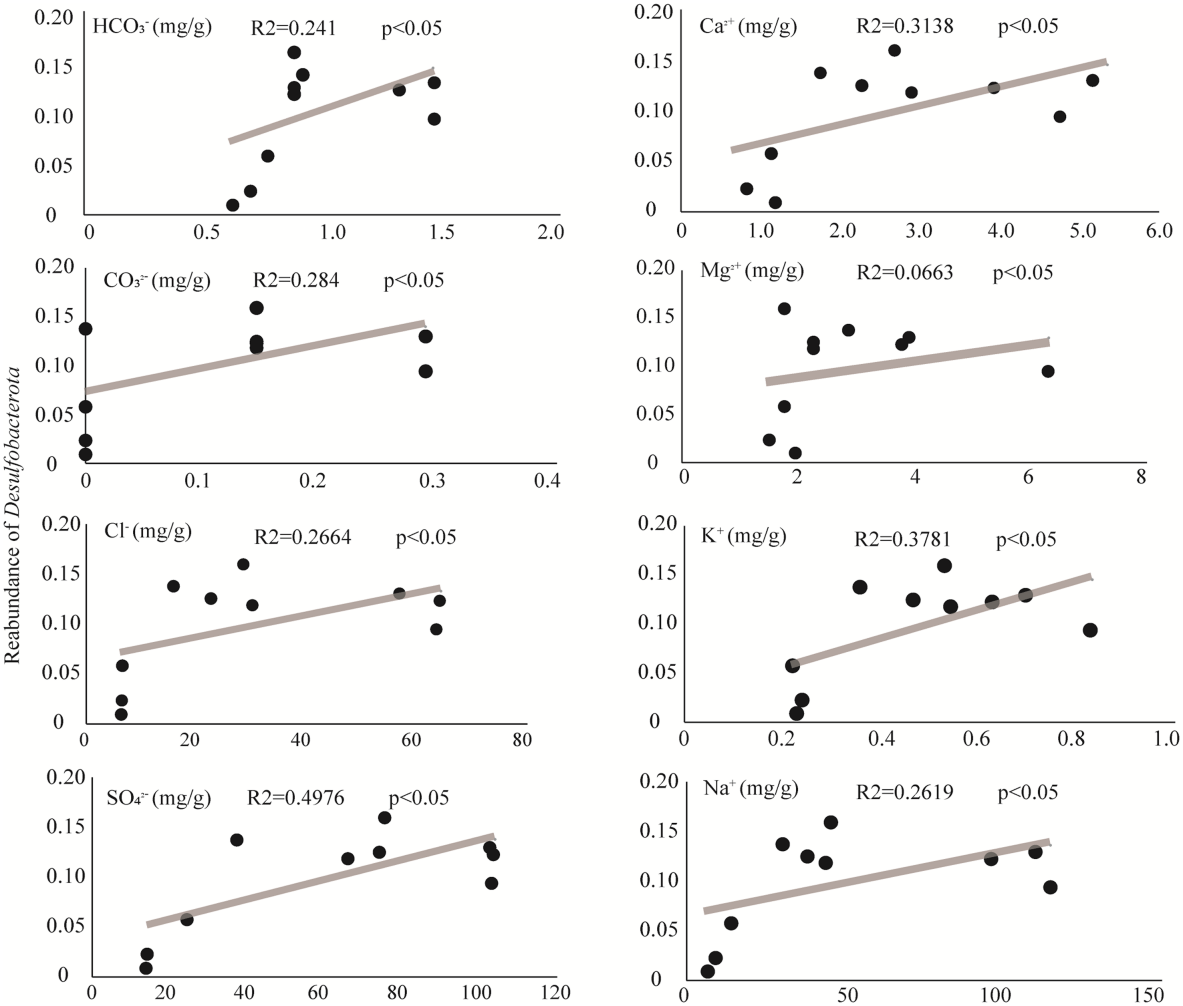
Correlating factors of *Desulfobacterota* from this study.

The phylum *Desulfobacterota* colonizes a variety of habitats of sediment, such as oceans, lakes, and rivers (Wei et al., 2023) with sulfate reduction characteristics. As a representative group in sulfate-type salty lake Barkol, it is important to explore the affecting factors of *Desulfobacterota*. The results of correlation analysis between environmental factors and relative abundance of the representative phylum *Desulfobacterota* in Barkol Saline Lake indicated that the correlation of this group with four major anions CO_3_^2−^, HCO_3_^−^, Cl^−^, and SO_4_^2−^, as well as four major cations Ca^2+^, Mg^2+^, K^+^, and Na^+^, was significantly positive (Figure 6). This indicated that *Desulfobacterota* might be a very important and representative group which plays a key role in the biogeochemical cycle of saline lake habitats, especially sulfur cycle coupled with other biogeochemical cycle.

### Function predicted by FAPROTAX

**Figure 7:**
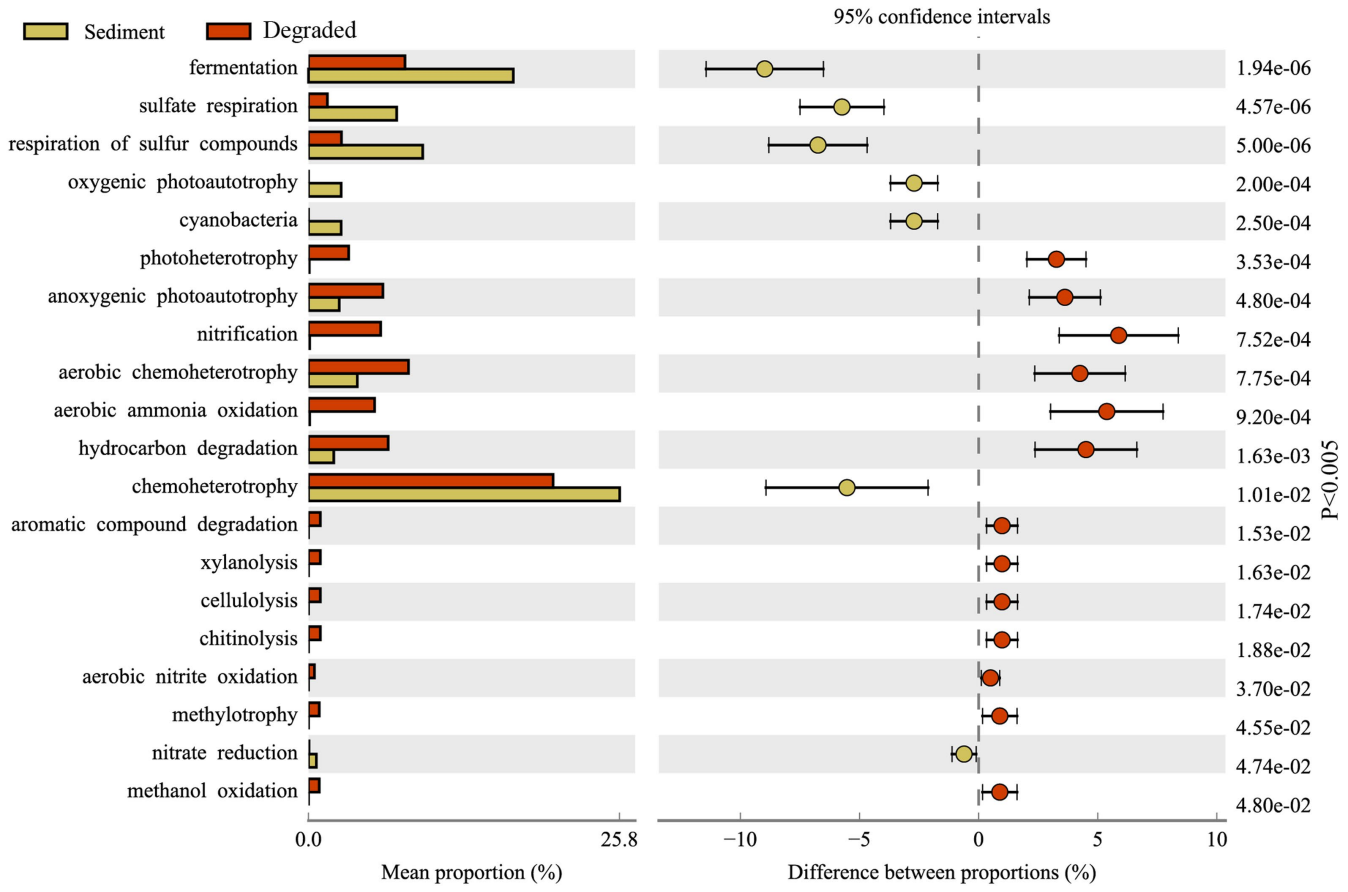
Variation of microbial functions from sediment samples and soil samples.

FAPROTAX has been proven to be a suitable tool for predicting the metabolic function of potential microbial communities. Figure 7 displays the FAPROTAX annotation that the predominant microbial functions in Barkol Lake habitat belonged to chemoheterotrophy. Overall, the FAPROTAX analysis revealed that the microbial communities in the Barkol Lake habitat were predominantly involved in chemoheterotrophy. In terms of the carbon cycle, fermentation was the most important metabolic pattern, and it was more abundant in sediment samples compared to degraded areas. In the sulfur cycle, the major procedure was the respiration of sulfur compounds, particularly sulfate respiration, which was much higher in sediment samples than in degraded areas. In contrast to the carbon and sulfur cycles, the nitrogen metabolism in soil samples from degraded areas was significantly higher, and this included processes such as nitrification, aerobic ammonia oxidation, and aerobic nitrite oxidation. Additionally, various metabolic procedures such as aromatic compound degradation, xylanolysis, cellulolysis, chitinolysis, aerobic nitrite oxidation, methylotrophy, and methanol oxidation were increased in the lake-degraded areas.

Through the functional prediction of this study, many microbial groups for basic element cycling were detected in the Barkol Saline Lake habitat (Supplementary Figure S3). The results further showed that the methylotrophy microbial community, including methanol oxidation, was only distributed in soil samples from degraded areas. However, oxygenic photoautotrophy represented by cyanobacteria was unique in sediment samples. The fermentation, organic matter was converted into acetic acid and hydrogen through acetogenesis, was much higher in sediment habitat. Respiration of sulfur compounds, fermentation, hydrocarbon degradation, and anoxygenic photoautotroph S oxidizing was widely distributed in all kinds of samples (Figure 7). The fact that xylanolysis, cellulolysis, and chitinolysis were only exhibited in soil samples means that xylan, cellulose, and chitin may not be effective carbon sources for microbial utilization in the sediment of Barkol Saline Lake. In the present study, manganese respiration, iron respiration, and other processes involved by microorganisms at Barkol Lake were also very important. Moreover, a key process, anammox, was also distributed at Barkol Lake from a degraded area. On the whole, the microbes from the saline Barkol Lake habitat were likely to participate in the processes of the biogeochemical cycle, such as carbon, nitrogen, and sulfur.

### Oxidative stress tolerant predicted by BugBase

The results from the prediction of BugBase showed that *Pseudomonadota* was the representative group for oxidative stress tolerance at the phylum level. *Thioalkalivibrio*, *Marinobacter*, and *Loktanella*, on the other hand, were representative genera of oxidative stress tolerance within this phylum, which are specifically known for their ability to tolerate stressful conditions (Supplementary Figure S4). Overall, microbial communities in degraded areas have a higher oxidative stress tolerance compared to those in sediments. This suggests that microorganisms in degraded areas have adapted to harsh conditions over time, making them more resilient in the face of stressors such as high salt, drought, and low temperature. These findings have important implications for understanding how microorganisms adapt and survive in different environments. Additionally, this research could be useful in developing stress-tolerant microbes for applications in environmental remediation and agriculture.

## Discussion

Saline lakes are highly sensitive to environmental changes, and climate change is expected to further exacerbate these effects (Gao et al., 2022). As an essential member of ecosystem, microorganisms are particularly important as they are the primary drivers of various biogeochemical processes (Li et al., 2023), such as nutrient cycling, energy transfer (Xie et al., 2022), carbon fixation (Berg et al., 2010; Hugler and Sievert, 2011; Xiao et al., 2023), and sulfur metabolism (Anantharaman et al., 2018; Zorz et al., 2019). However, the degradation of inland lakes, such as Barkol Saline Lake, has been a serious environmental problem in recent years. The stability of community structure and complex functions could maintain the sustainability and integrity of different ecosystems on earth (Gao et al., 2023). Therefore, understanding the dynamic change of microbial communities and their metabolic functions in degraded saline lake environments is important.

### The microbial community diversity was significantly decreased in the process of lake degradation

In the present research, the microbial community diversity was significantly reduced in the degraded areas compared to the sediment samples, which had a higher level of diversity and consistency. These results indicate that the number and types of microorganisms present in the degraded area were decreased, leading to deterioration or decline in the overall health and functioning of the lake ecosystem. According to previous research, the community structure refers to the composition and organization of different species within an ecosystem. There is a balance and interdependence among different species in a healthy ecosystem (Simachew et al., 2016; Han et al., 2017). However, the degradation of saline lakes disrupts this balance, leading to changes in the community structure.

Additionally, the microbial community composition and structure showed that the abundance of ASV belonging to *Desulfobacterota* decreased as the lake sediment degraded into soil. Meanwhile, the results of correlation analysis between environmental factors showed that *Desulfobacterota* was positively correlated with four major anions: CO_3_^2−^, HCO_3_^−^, Cl^−^, and SO_4_^2−^, as well as four major cations: Ca^2+^, Mg^2+^, K^+^, and Na^+^. This could be due to the fact that *Desulfobacterota* are known to be adapted to hypersaline habitats (Liu et al., 2023). This group (sulfate reduction) includes 15 strains belonging to two novel genera, *Desulfonatronum* and *Desulfonatronovibrio* (Sorokin et al., 2011), a haloalkaliphilic heterotrophic SRB (Sorokin et al., 2012), and an acetate-oxidizing SRB with extremely salt-tolerant properties from hypersaline soda lakes (Sorokin et al., 2015). With the concentration of sulfate decreasing, the microbial community may exhibit two ecological adaptation strategies: one is changing their respiration or energy metabolic pathway, and another is enabling different SRB communities (Varon-Lopez et al., 2014). Certainly, further study is necessary for the verification of specific functionalities, such as sulfate reduction, under laboratory conditions. Moreover, the saline lake habitat, with its extreme conditions such as high salinity (Tang et al., 2012), low oxygen levels (Cypionka, 2000), and high UV radiation (Jookar Kashi et al., 2021), provides a unique environment for microbial life. The discovery of many unclassified microbial groups in this study suggests that there is still much to learn about the diversity and function of microorganisms in saline lake environments.

### Microbial community was more stable in sediment than in degraded areas

The microbial community assembly in Barkol Saline Lake ecosystems was predominantly shaped by deterministic processes. This means that the composition and structure of the microbial communities were mainly influenced by environmental factors and niche selection (Gao et al., 2022). However, at the boundary area between the sediment and degraded areas, the community assembly seemed to be undergoing a stochastic process, which implies that random factors, such as dispersal and chance events were important. Salinity as a special environment factor was always considered to be a critical factor (Yang et al., 2016) and influenced microbial communities in different niches (Liu et al., 2016), especially in saline lakes with higher salty concentrations (Rath et al., 2019). To determine the factors influencing microbial distribution, we conducted further analysis of the physicochemical properties of the samples. It revealed that the concentration of major ions exhibited a decreasing trend after lake degradation. This indicates that the overall concentration of salt ions decreases as the lake degrades, which will eventually lead to a decrease in total salinity and inevitably change the corresponding microbial groups in this habitat (Gao et al., 2022). The co-occurrence network patterns revealed that the complexity of microbial community in the Barkol Saline Lake habitat was: all>sediment>degraded area. This further indicates that lake degradation will first lead to the reduction of microbial species and diversity, and second, it will further reduce the stability of community structure, which is not conducive to the protection and maintenance of the saline lake habitat (Marois et al., 2022).

### The sulfur metabolism was reduced in the degraded areas compared to the sediment

Strikingly, the degradation process of Barkol Saline Lake sediments changed the selective pressure. For functional groups, the abundances of some microbial communities were correlated to their ecological function in the ecosystem. Our findings, as well as previous studies, have all shown that fermentation was the most important metabolic pattern in sediment samples (Huang et al., 2020; Liu et al., 2023; Lomakina et al., 2023). The reason for this could be illustrated by the fact that the saline lake sediment is an anaerobic environment that lacks oxygen while being rich in organic matter (Wasmund et al., 2017). These microorganisms obtain their energy through fermentation, which could produce a variety of compounds as byproducts, including organic acids, alcohols, methane, and other metabolites (Anantharaman et al., 2018). Additionally, various metabolic procedures such as aromatic compound degradation, xylanolysis, cellulolysis, chitinolysis, aerobic nitrite oxidation, methylotrophy, and methanol oxidation were increased in the degraded areas. These findings suggest that the microbial communities in Barkol Lake habitat undergo significant changes in metabolic function as the lake becomes degraded. The increased nitrogen metabolism in degraded areas may be a response to changes in nutrient availability, while the increased metabolic procedures related to the degradation of organic compounds may be a result of increased organic matter input in the degraded areas. The predicted prevalence of sulfur metabolism genotypes, particularly sulfate respiration genotypes, was much higher in sediment samples compared to degraded areas. However, climate change has aggravated the degradation of the saline lake in recent decades, and the salt composition of sediments has also gradually changed with outside conditions (Marois et al., 2022). In Barkol Lake, the main salt was sulfate compounds such as sodium sulfate or gypsum; hence, sulfur-related microorganisms were long-established functional groups. Besides, the microbial communities in degraded areas have a higher stress tolerance compared to those in sediments. This suggests that microorganisms in degraded areas have adapted to harsh conditions over time, making them more resilient in the face of stressors such as high salt, drought, and low temperature (Tang et al., 2012; Lu et al., 2013).

## Data availability statement

The datasets presented in this study can be found in online repositories. The names of the repository/repositories and accession number(s) can be found in the article/Supplementary material.

## Author contributions

Y-HL: Data curation, Funding acquisition, Methodology, Project administration, Writing – original draft, Writing – review & editing. LG: Resources, Software, Writing – review & editing. H-CJ: Writing – review & editing, Writing – original draft. B-ZF: Writing – review & editing, Resources. YH: Writing – review & editing, Resources. LL: Writing – review & editing, Resources. SL: Writing – review & editing, Methodology, Software. RA: Writing – review & editing, Software. W-HL: Writing – review & editing, Software. J-YZ: Writing – review & editing, Software. Z-DY: Software, Writing – review & editing. OM: Writing – review & editing, Resources, Writing – original draft. W-JL: Resources, Supervision, Writing – review & editing.

## Footnotes

^1^ https://qiime2.org/

## Supplementary material

### SUPPLEMENTARY FIGURE S1

The rarefaction curves **(A)** and relative abundance **(B)** from sediment and degraded area samples.

### SUPPLEMENTARY FIGURE S2

The relationship of community distribution from sediment and degraded area samples drawn by Circos diagram.

### SUPPLEMENTARY FIGURE S3

The microbial functions from sediment samples and soil samples predicted by FAPROTAX.

### SUPPLEMENTARY FIGURE S4

The variation of microbial stress tolerant from sediment samples and soil samples at the phylum **(A)** and genus level **(B)**, respectively.
