# Supplementary figures and images for: Response of microbial diversity and function to the degradation of Barkol Saline Lake
Source: Front Microbiol. 2024 May 9;15:1358222. doi: 10.3389/fmicb.2024.1358222 (PMC11111964; doi:10.3389/fmicb.2024.1358222)

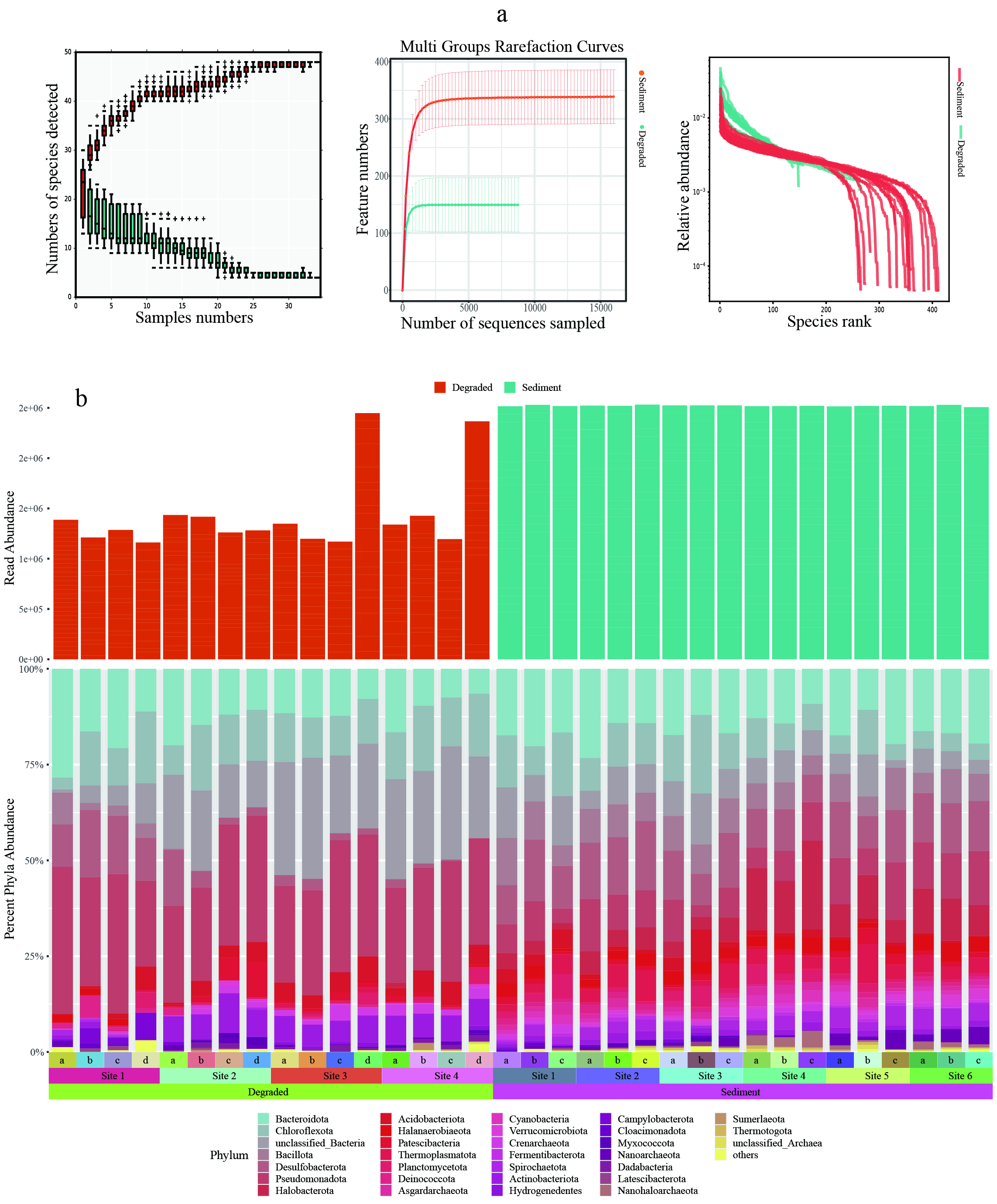

Supplement: Supplementary file 1 [file Image_1.JPEG]

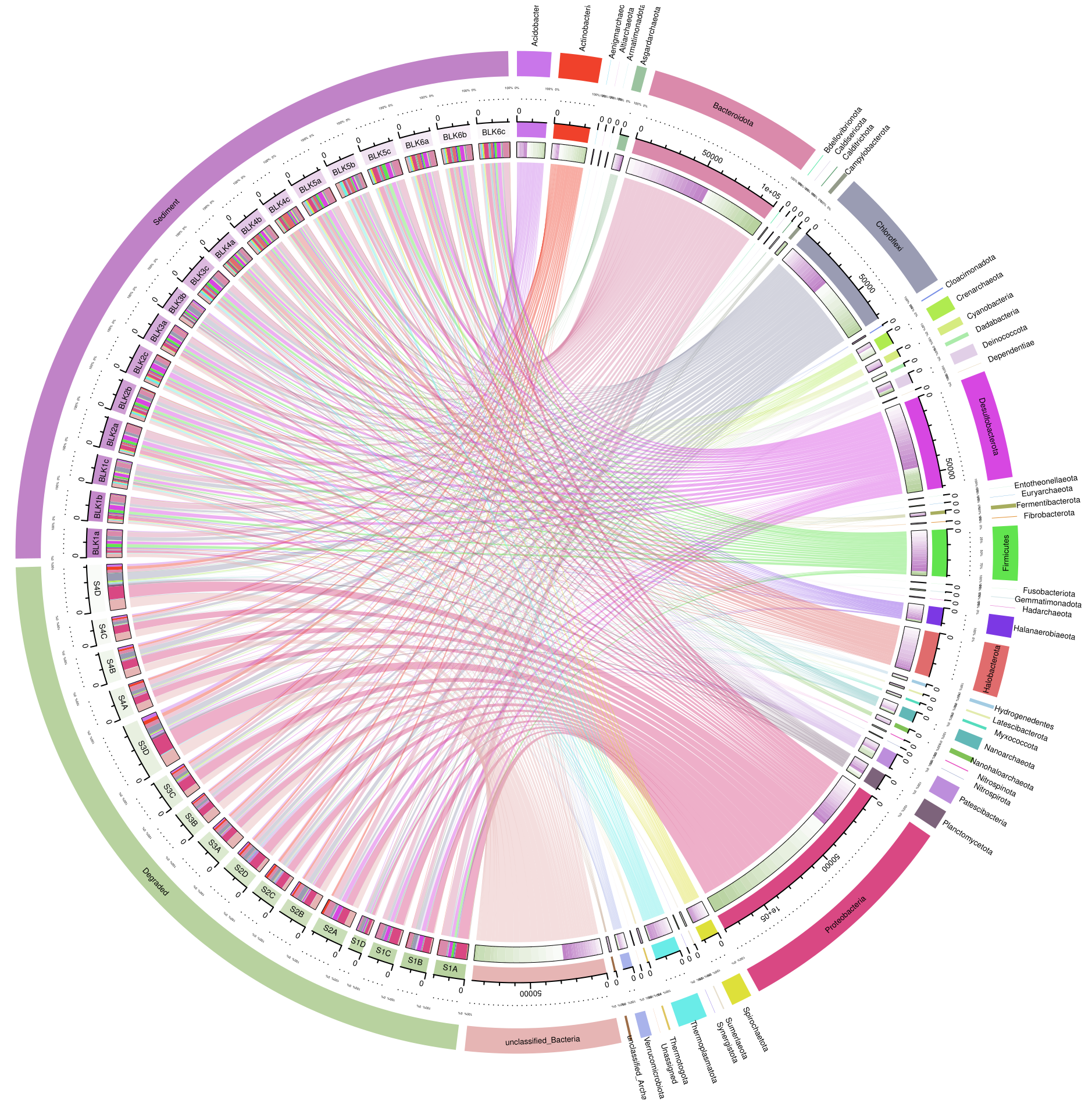

Supplement: Supplementary file 2 [file Image_2.TIF]

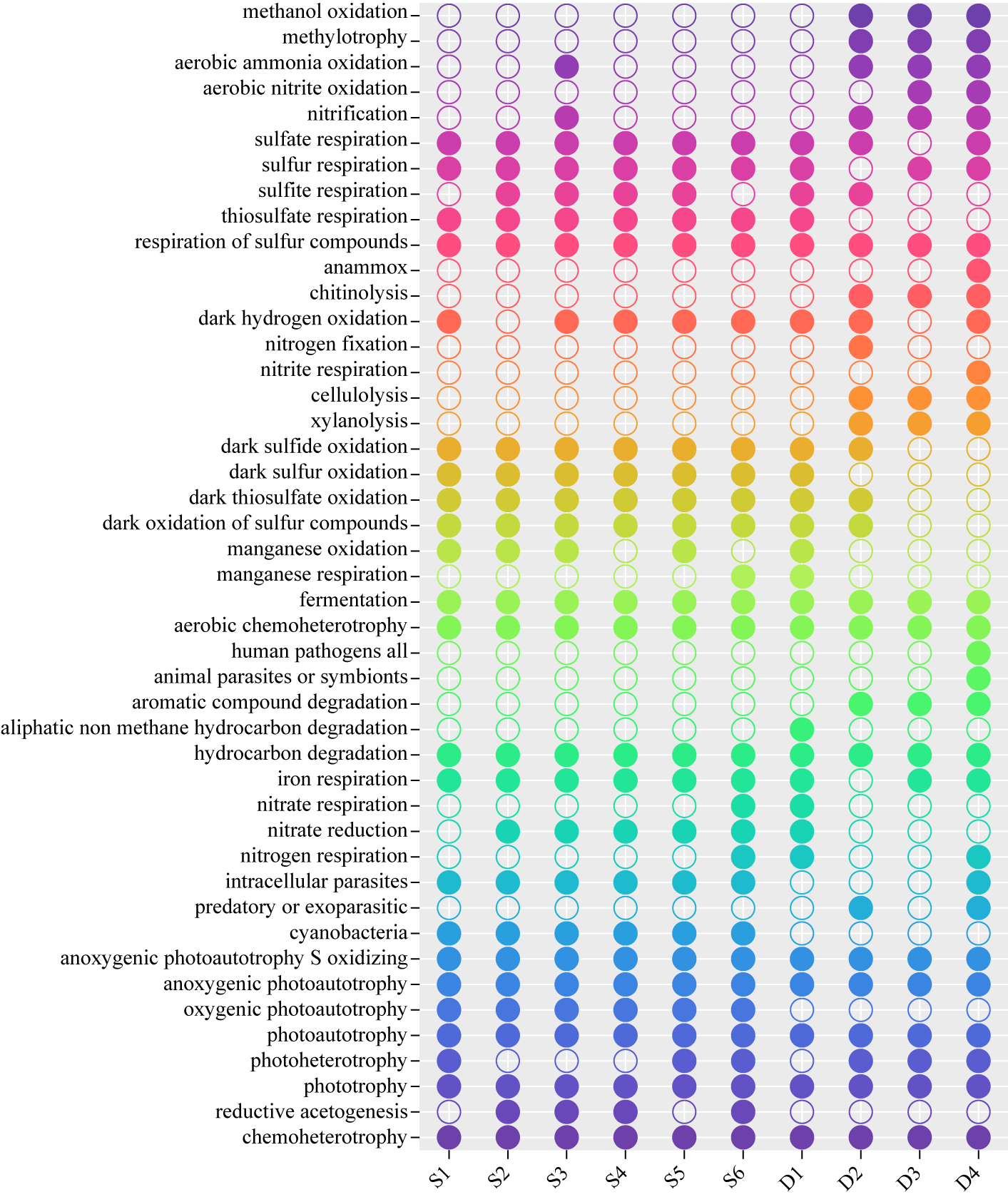

Supplement: Supplementary file 3 [file Image_3.TIF]

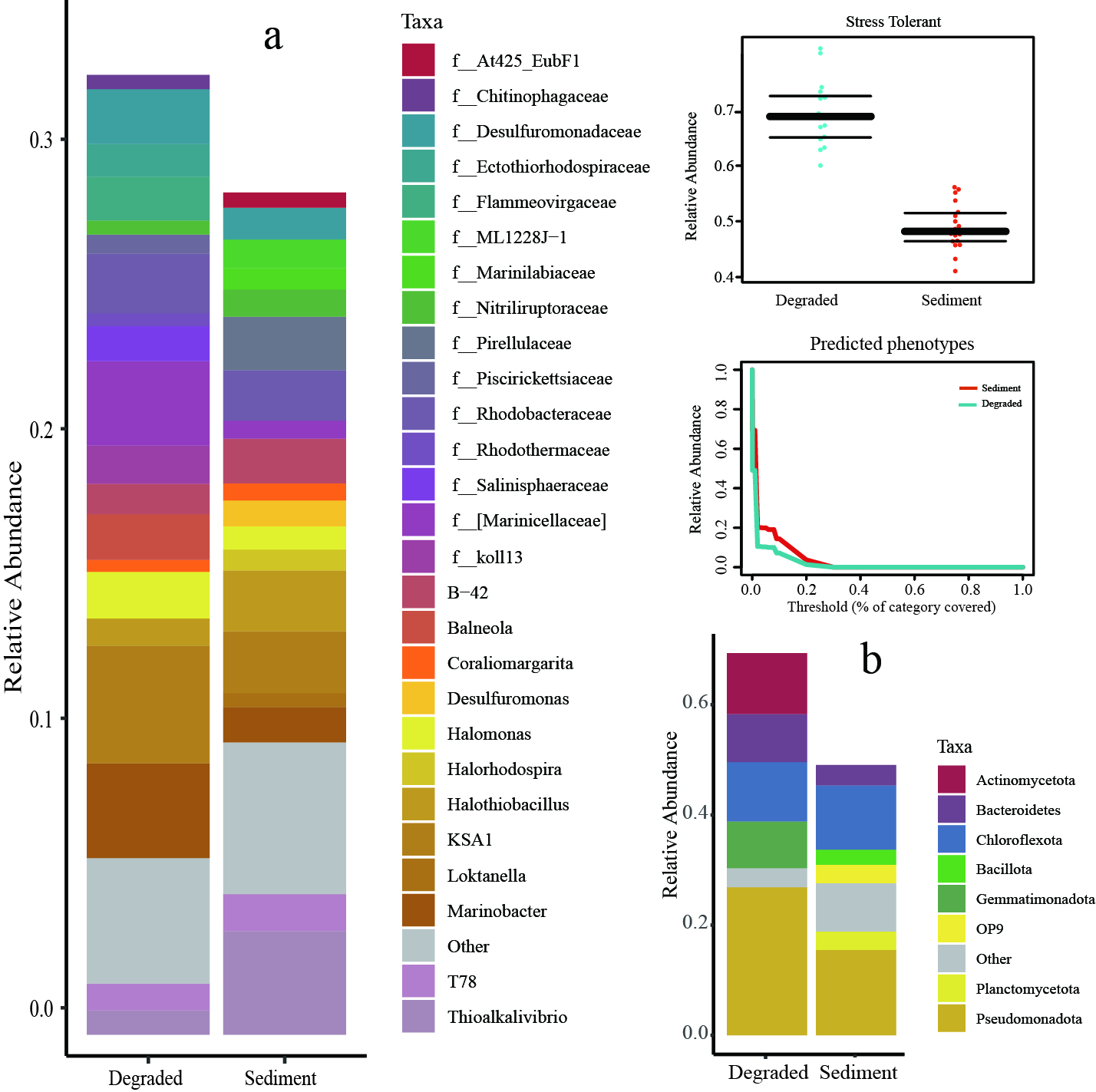

Supplement: Supplementary file 4 [file Image_4.JPEG]
